# Exploring the Embodiment of a Virtual Hand in a Spatially Augmented Respiratory Biofeedback Setting

**DOI:** 10.3389/fnbot.2021.683653

**Published:** 2021-08-27

**Authors:** Giacinto Barresi, Andrea Marinelli, Giulia Caserta, Massimiliano de Zambotti, Jacopo Tessadori, Laura Angioletti, Nicolò Boccardo, Marco Freddolini, Dario Mazzanti, Nikhil Deshpande, Carlo Albino Frigo, Michela Balconi, Emanuele Gruppioni, Matteo Laffranchi, Lorenzo De Michieli

**Affiliations:** ^1^Rehab Technologies, Istituto Italiano di Tecnologia, Genoa, Italy; ^2^Department of Informatics, Bioengineering, Robotics, and Systems Engineering, Università degli Studi di Genova, Genoa, Italy; ^3^Movement Biomechanics and Motor Control Lab, Department of Electronics, Information and Bioengineering, Politecnico di Milano, Milan, Italy; ^4^Center for Health Sciences, SRI International, Menlo Park, CA, United States; ^5^Visual Geometry and Modelling, Istituto Italiano di Tecnologia, Genoa, Italy; ^6^International Research Center for Cognitive Applied Neuroscience, Università Cattolica del Sacro Cuore, Milan, Italy; ^7^Research Unit in Affective and Social Neuroscience, Department of Psychology, Università Cattolica del Sacro Cuore, Milan, Italy; ^8^Advanced Robotics, Istituto Italiano di Tecnologia, Genoa, Italy; ^9^Centro Protesi INAIL, Istituto Nazionale per l'Assicurazione contro gli Infortuni sul Lavoro, Bologna, Italy

**Keywords:** embodiment, augmented reality, prosthetics, biofeedback, training, breathing

## Abstract

Enhancing the embodiment of artificial limbs—the individuals' feeling that a virtual or robotic limb is integrated in their own body scheme—is an impactful strategy for improving prosthetic technology acceptance and human-machine interaction. Most studies so far focused on visuo-tactile strategies to empower the embodiment processes. However, novel approaches could emerge from self-regulation techniques able to change the psychophysiological conditions of an individual. Accordingly, this pilot study investigates the effects of a self-regulated breathing exercise on the processes of body ownership underlying the embodiment of a virtual right hand within a Spatially Augmented Respiratory Biofeedback (SARB) setting. This investigation also aims at evaluating the feasibility of the breathing exercise enabled by a low-cost SARB implementation designed for upcoming remote studies (a need emerged during the COVID-19 pandemic). Twenty-two subjects without impairments, and two transradial prosthesis users for a preparatory test, were asked (in each condition of a within-group design) to maintain a normal (about 14 breaths/min) or slow (about 6 breaths/min) respiratory rate to keep a static virtual right hand “visible” on a screen. Meanwhile, a computer-generated sphere moved from left to right toward the virtual hand during each trial (1 min) of 16. If the participant's breathing rate was within the target (slow or normal) range, a visuo-tactile event was triggered by the sphere passing under the virtual hand (the subjects observed it shaking while they perceived a vibratory feedback generated by a smartphone). Our results—mainly based on questionnaire scores and proprioceptive drift—highlight that the slow breathing condition induced higher embodiment than the normal one. This preliminary study reveals the feasibility and potential of a novel psychophysiological training strategy to enhance the embodiment of artificial limbs. Future studies are needed to further investigate mechanisms, efficacy and generalizability of the SARB techniques in training a bionic limb embodiment.

## Introduction

Artificial limbs are designed to assist and increase the manipulation capabilities of human beings in contexts from teleoperation to virtual rehabilitation, to bionic prosthetics (Makin et al., 2020). In order to nurture the progress of this research domain, scientists considered the results of studies on topics like the proprioceptive illusions in people with a spinal cord injury (Fusco et al., 2016) or the applications of error-related potentials in neuroprosthetics (Iturrate et al., 2015). Through the integration between neuroscience and engineering, interdisciplinary research has offered inspiring strategies like developing neurointerfaces to control virtual and robotic systems (Tidoni et al., 2016) or neuromorphic systems to bring the sense of touch to the prosthesis users (Rongala et al., 2018).

Artificial limbs can be perceived by certain users as tools, while others can feel them as corporeal structures (Murray, 2004). In this second case, these robotic or virtual extensions of the user can trigger the phenomenon of embodiment, i.e., the psychological process occurring when subjects feel external objects as integrated in their own body scheme (Mor and Makin, 2020).

However, the embodiment process is not limited to artificial limbs, and can involve any artifact or tool (Pazzaglia and Molinari, 2016). Initially, this process makes the device more familiar for the users who have become curious about it. Subsequently, the mental representations of the users start to adjust to progressive human-artifact integration (Nelson et al., 2020). Feeling a device as embodied leads to improvements in user's engagement, technology acceptance, control transparency, and, consequently, human-machine system performance (Toet et al., 2020).

Typically, the investigations in this domain aim at establishing effective methods to enhance the embodiment through the manipulation of the stimulus-conditions (Ratcliffe and Newport, 2017) or the active control conditions of artificial limbs (Brugada-Ramentol et al., 2019). However, literature on interoceptive processes (Allen and Tsakiris, 2018) suggests that an individual's psychophysiological control potentially impacts on embodiment components like body ownership. It is hypothesized that respiratory entrainment techniques (Czub and Kowal, 2019) like those used in contemplative mental training and biofeedback (Bornemann, 2017), may influence the embodiment process.

This paper preliminarily investigates whether modulating one's psychophysiological state via respiratory biofeedback can enhance the embodiment of a virtual, computer-generated hand. Our research was carried out through a pilot study using common devices like a computer monitor, a smartphone, and a microphone. This last choice was made to explore the potential of a setup that can be replicated at home without the need for special equipment. Evaluating the feasibility of this setup is our second scope for extending the upcoming data collection (bypassing also the restrictions of the current pandemic) (Woolliscroft, 2020) through this innovative “embodiment training” approach.

## Background and Scope

### Related Works

As hinted above, several studies on embodiment (Niedernhuber et al., 2018) aim at improving human-machine interaction with special attention to artificial limbs user experience, especially to reducing prosthetic devices abandonment (Beckerle et al., 2019) and promoting their acceptance and integration (Shaw et al., 2018). Indeed, the results of embodiment studies are quite helpful to guide the design of novel artificial limbs through an improved understanding of user experience: a survey involving 2,383 limb amputees highlighted how naturalistic prostheses designed with sensory feedback were associated with higher feeling of prosthesis ownership and reduced phantom pain (Bekrater-Bodmann et al., 2021).

According to literature (Toet et al., 2020), the sensations of ownership (the feeling that non-bodily objects are body parts and sources of bodily sensations, depending on the integration of multisensory inputs), self-location (the feeling of the body location in space, depending on the co-location of fake and real elements), and agency (the feeling of being the cause and the author of observed actions, depending on the efficiency of limb motor control) constitute the embodiment (Kilteni et al., 2012) process itself.

Considering the case of artificial upper limbs, the investigation of their embodiment is usually entrusted to methods for evaluating a well-known phenomenon that demonstrated high potential in experimental and clinical neuroscience research (Ramakonar et al., 2011): the Rubber Hand Illusion (RHI) (Botvinick and Cohen, 1998). RHI is typically induced by the co-occurrence of visual stimulations on an inactive fake limb observed by the subjects and tactile stimulations on their real hand (Kammers et al., 2009).

In particular, RHI studies offer important pointers toward investigating the ownership component of embodiment. The body ownership is especially critical in the acceptance of artificial limbs—see Ehrsson et al. (2008) and Beckerle et al. (2018). This aspect of the embodiment was investigated through multiple studies, considering, for instance, its relationships with sensory stimulations (Ehrsson et al., 2005) and other embodiment components—agency (Tsakiris et al., 2006) and self-location (Romano et al., 2015). Interestingly, RHI can also generate phenomena of disembodiment as the disownership of the hidden real hand (Lewis and Lloyd, 2010). These and other seminal studies have contributed to the research in this area, which embraced topics like the impact of affective processes (Crivelli and Balconi, 2020) or the psychopathological aspects (Prikken et al., 2019) in body ownership representations.

These are just examples of the body ownership literature, which is rich with original methodological solutions to assess how this phenomenon occurs in different conditions. Overall, the body ownership is typically evaluated in RHI paradigms through measures like subjective evaluations (e.g., self-report questionnaires) (Romano et al., 2021) or physiological reactions (e.g., Galvanic Skin Response, GSR) (Grechuta et al., 2017). Another classic measure of ownership is the proprioceptive drift (Tsakiris and Haggard, 2005) toward the artificial limb when the subjects are asked to estimate the actual position of their own hand, usually hidden and apparently replaced by a fake one during the experimental session. This implicit measure is performed in different ways according to the experimental setting—e.g., a virtual version in Ma et al. (2021).

It must be noted that the use of such body ownership measures in RHI studies is still debated: for instance, distinctions between subjective questionnaire scores and proprioceptive drift (Gallagher et al., 2021) should be further investigated to understand different processes underlying the subjective evaluation and the proprioception.

Alongside the research on the heterogenous manifestations and measures of the ownership, literature has also shown structured models to understand its role within the bodily representations. According to Tsakiris (2010), body ownership depends on the interplay between the current multisensory input (bottom-up processes) and the internal models of the body (top-down modulation) that phenomenologically lead to conditions like the RHI (Tsakiris and Haggard, 2005). Specifically, the malleability of bodily representations can depend on interoception (Herbert and Pollatos, 2012), the perception of the internal state of the body. In particular, individuals with low interoceptive sensitivity (assessed through a heartbeat monitoring task) experience a stronger illusion of ownership in RHI (Tsakiris et al., 2011).

Within this research domain, typical methodologies based on purely exteroceptive visuo-tactile stimulations tend to be substituted by combinations of interoceptive and exteroceptive signals, like cardio-visual stimulations (Allen and Tsakiris, 2018). For instance, observing a virtual hand that is pulsating in synchrony with participant heartbeat can induce body ownership changes as reported in RHI experiments (Suzuki et al., 2013). Other studies investigated heartbeat-evoked electroencephalographic (EEG) potentials and their role in bodily self-consciousness (Park et al., 2018).

The role of interoceptive sensitivity in RHI was also investigated in Xu et al. (2018). Specifically, authors studied the effects of meditation and mindfulness practices—like respiratory control or heartbeat control—on RHI susceptibility. Authors highlighted how meditators subjectively rated the RHI less strongly than non-meditators. These results are coherent with the ones of Cebolla et al. (2016) on the agency perceived by meditators in RHI, and with Tsakiris et al. (2011). However, in Xu et al. (2018), no difference in proprioceptive drift was found between these meditators and non-meditators, and different interoceptive awareness factors were associated with RHI intensity in meditators. Thus, it can be inferred that practicing meditation could lead to different embodiment experiences when subjected to an interoceptive training to flexibly shift attention along the body; it makes the person more resistant to abnormal sensations.

This conclusion suggests the possibility that our malleable body representations could be affected by meditation exercises. However, the evidence in Xu et al. (2018) was based on a typically passive RHI procedure executed by people who previously practiced meditation techniques. The prior meditation experience had, apparently, shaped the people's interoceptive sensitivity and body awareness before any RHI experience. This led us to a question: how could certain exercises practiced in meditation affect the embodiment of an artificial limb if they directly contribute to making an artificial limb illusion happen? An answer to this question could lead to novel approaches of embodiment training based on active self-regulation techniques that assist the artificial limb ownership.

In the current study, we targeted a core component of meditation practice, i.e., the breathing (Brenner et al., 2020), particularly slow breathing, which is commonly performed at 6–10 breaths per min. Slow paced breathing produces multiple psychophysiological changes (Zaccaro et al., 2018), characterized by a generalized relaxation across, for instance, cardiovascular and cortical domains, especially with regard to meditation (Yu et al., 2018). Overall, this respiratory exercise has pervasive effects on autonomic functions, downregulating them (Russo et al., 2017). Furthermore, these effects can involve the interoceptive awareness (Weng et al., 2021) through a self-regulation that is relatively easy for a practitioner. Here, we considered respiratory biofeedback (targeting 0.1 Hz respiratory rate)—self-modulating the Respiratory Rate (RR) according to its visualization (Blum et al., 2020)—for its effectiveness in influencing the physical and mental states has been shown in literature (de Zambotti et al., 2019).

In order to proceed with our investigation, we decided to adopt a promising approach for exploring embodiment processes like the body ownership through an interactive solution with high perceptual versatility: the Virtual Hand Illusion (VHI) (Raz et al., 2008). VHIs are produced through a setup that offers a complete experimental control of engaging computer-generated scenarios (Milgram and Kishino, 1994) of Virtual Reality (VR—where the perceptual scenario is fully generated by a computer) or Augmented Reality (AR—where virtual items are placed within a real perceptual scene) or Mixed Reality (MR—where virtual items and real items co-exist, often emphasizing the possibility to interact with the first ones as physical objects, according to some authors) (Speicher et al., 2019). Overall, these systems can be considered as cases of Extended Reality (XR), which is becoming a trend in neuroscientific research as well (Parsons et al., 2020).

Thanks to their versatility in controlling the perceptual scene (Tieri et al., 2017) and to their capability to motivate and engage the subjects through game-based features (Škola et al., 2019), XR systems offer fertile opportunities for body ownership studies as demonstrated by IJsselsteijn et al. (2006) and Slater et al. (2008)—for a review on this topic, read Škola and Liarokapis (2016). Such solutions, extremely valuable in clinical applications too (Matamala-Gomez et al., 2021), demonstrate further potential through their compatibility with other technologically advanced approaches like neuromodulation (Kannape et al., 2019). Furthermore, AR solutions are currently explored to train the control of prosthetic systems (Boschmann et al., 2021).

Interestingly, the study in Monti et al. (2020) adopted a VR-based RR biofeedback approach to generate and investigate an “embreathment” illusion by ecologically mapping the subjects' breaths onto a virtual body observed from a first-person perspective, improving the embodiment of the individual on the avatar. The authors highlight the potential of breathing as a natural, continuous, multisensory self-stimulation. Furthermore, they demonstrate the opportunity of implementing such a self-regulation process through an engaging virtual environment.

Summing up, XR settings can be exploited to investigate the effects of a slow respiratory biofeedback exercise as a method to enhance the embodiment of an artificial limb.

### Research Objectives

Our hypothesis is that slow respiratory biofeedback, as a self-regulation strategy, can facilitate the embodiment of a virtual hand during a biofeedback training designed to evoke a VHI. Accordingly, this pilot study aimed at comparing two conditions of respiratory biofeedback—slow breathing and normal breathing—in terms of indices of virtual hand ownership sensation. We considered an interactive setup that enables the person to control the perceptual features of a computer-generated hand without moving it (as in typical RHI and VHI). This allows us to focus on the body ownership component of embodiment as a premise for further studies.

Through this proof of concept, we also investigated the feasibility of a protocol designed for remote use, which only requires a computer, a microphone, a monitor, and a smartphone. If successful, this would provide a portable and affordable solution to enable anyone (for example an amputee waiting to receive a prosthesis) to perform at home a novel biofeedback-enhanced embodiment training. This choice was also driven by the need of creating a remote version of this setup for upcoming studies due to the limitations imposed by COVID-19 (e.g., stay home orders).

## Materials and Methods

### Participants

All participants were volunteers from IIT, signed the informed consent and followed the IIT ADVR TELE01 experimental protocol approved (on March 16th, 2020) by the Ethical Committee of Liguria Region in Genoa, Italy. Before recruiting the participants, the sample size was calculated through G^*^Power v3.1.9.7 (Faul et al., 2007) according to the results of preparatory tests (involving eight subjects) performed to improve the user-centered design of the setup. These results were based on the differences between two conditions in mean (−2.75) and standard deviation (4.36) of proprioceptive drift scores (see Intructions and Tasks) compared through paired samples *t*-test (more restrictive in terms of requirements than non-parametric tests used for other measures like questionnaire scores). Thus, with α = 0.05, power = 0.8, G^*^Power estimated a sample size of 22 subjects.

Twenty-two (six females) adults (Age, mean ± SD: 27.4 ± 2.4 years) without disabilities participated in the study. Twenty subjects were right-handed, one subject was left-handed, one subject was ambidextrous. Only two subjects declared to have had respiratory difficulties (respectively moderate asthma and rhinosinusitis) in past. All individuals were free from sensory and cognitive disabilities, and motor impairments derived from neurological conditions, and psychoactive drugs consumptions in previous 6 months. To avoid any potential RHI-resistance of meditators (Xu et al., 2018), all participants were selected as naïve about mindfulness and meditation techniques.

To assess how prosthesis users could approach this kind of task with the proposed setup within an embodiment training protocol, two (66 and 33 years old) male amputees (users of transradial prostheses for the right upper limb) without respiratory issues were also recruited and performed the same procedure as the 22 participants described above, except for the biosignal data collection (simulating the home setting).

### Experimental Setup

**Figure 1:**
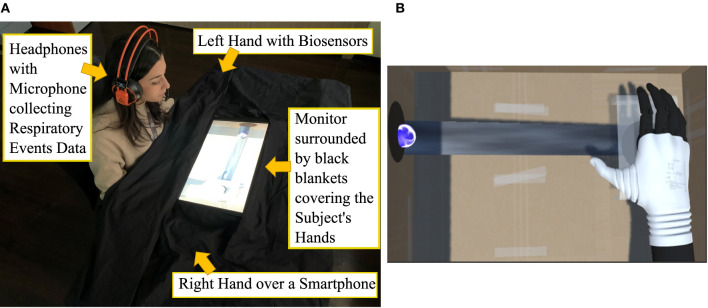
Experimental setting with **(A)** participant and **(B)** scene on the display.

All experimental sessions took place at Istituto Italiano di Tecnologia (IIT—Genoa, Italy). However, to design a setup compatible with upcoming home-based data collection, we did not use any head-mounted display typically adopted in highly immersive VHI settings with advanced haptic feedback systems (Beckerle, 2021). Thus, we considered the options offered by Spatial Augmented Reality (SAR) (Raskar et al., 1999) environments, where the real world is enriched by displays (including projections) placed across the real setting instead of being worn by the user as in the most typical AR paradigms based on visors (Bimber and Raskar, 2019). In our case, a computer monitor became a screen-based display for SAR. The final setting (Figure 1) was constituted by basic equipment available to anyone at home (monitor, smartphone, headphones) with the addition of professional systems for recording biosignals.

**Figure 2:**
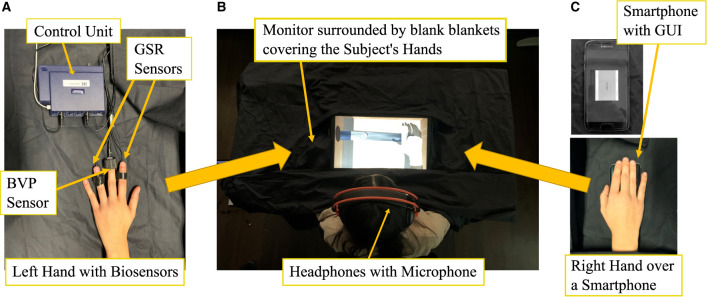
Experimental setup. **(A)** Physiological recording equipment. **(B)** Display and headset. **(C)** Vibratory feedback device.

To use the setup, the participants (Figure 1A) were positioned in front of a monitor (21” with 16:9 ratio, laid-out horizontally, slightly tilted toward them), wearing a headset with a microphone placed in front of their mouth. Black blankets covered the subjects' arms and surrounded the monitor to make the subject focus on the non-immersive virtual scenario presented by the screen (Figure 1B)—for the same reason, during the experimental session the environmental light was dim. A laptop (Alienware M15; Windows 10 Home 64 bit) was used to perform real-time processing of the audio data and extract breathing information used for visuo-tactile biofeedback. All participants wore photoplethysmography sensors to collect Blood Volume Pressure—BVP—data (providing a second estimation of breathing events, thus the RR in Hz, in respect to our custom microphone-based system) and skin conductance sensors collecting GSR data (source of potential embodiment-related reactions, expressed in μS) on left hand fingers. Specifically (Figure 2A), the BVP sensor was placed on the middle finger, the GSR sensors—Ag/AgCl electrodes mounted without conductive gel as in Visnovcova et al. (2020)—were placed on the middle phalanges of the index finger and the ring finger as in Gümüslü et al. (2020).

These sensors constituted an acceptable compromise to record biosignals without excessively altering the individual experience (this reason led to exclude the use of a chest belt). All biosensors were connected to the FlexComp Infiniti control unit (Figure 2A), connected to the laptop enabling the SAR scene (Figure 1B) and setting (Figure 2B). A smartphone (Samsung S7) for vibratory stimulations was placed under their right hand (Figure 2C).

Coherently with the SAR concept, this setting showed a continuity between the subjects' body and the virtual hand presented by the display, just like a prosthesis would replace a missing limb or a rubber hand would be placed in the typical RHI studies. Specifically, the screen presented an interactive environment developed in a Unity (https://unity.com) game project comprising 13 scenes per experimental condition.

This environment represented the inside of a cardboard box containing, on the right half, the 3D model of the Hannes (Laffranchi et al., 2020) prosthetic hand (Figure 1B). The choice of using the model of an actual prosthesis was made to allow for upcoming comparisons with real settings including the actual Hannes system in RHI-like studies. The hand model was created with the 3D design program Blender (https://www.blender.org) starting from single-part STL files of the Hannes prosthetic hand to preserve the real joint axes and related joint movements of the human hand. The Blender object was, then, imported in the Unity scenes, maintaining the properties of its different parts.

Inside the virtual cardboard box, a blue sphere “made of energy” (an engaging game-like design imported from a Unity package: ArtStation—Glowing orbs VFX, Vladyslav Horobets) slid from left to right on an inclined surface, coming out from a hole on the left side of the box. In 1 min, the sphere reached a black area (designed to magnify the position of the trial goal) with a hole placed under a right virtual hand, leant on a support that represents the presence of the smartphone under the real limb of the subjects. A hole through the virtual support enables the “contact” between the computer-generated hand and sphere. Figure 1B depicts the scene.

**Figure 3:**
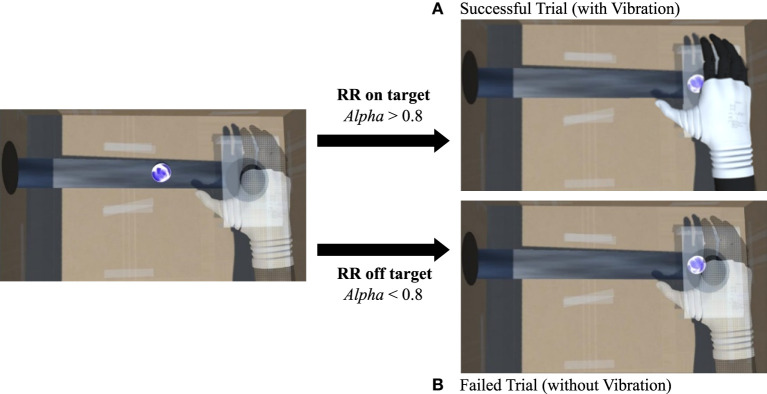
Examples of **(A)** successful trial (the sphere reaches the virtual hand in fully visible state) and of **(B)** failed trial (the sphere reaches the virtual hand in transparent state).

This SAR setting was then enriched by respiratory biofeedback features (based on RR data collected through the microphone of the earphones) within a Spatially Augmented Respiratory Biofeedback —SARB—paradigm. In this SARB implementation, the subjects modulated their own RR according to a target frequency in order to minimize the transparency (managed through a Unity package: Unity Stipple Transparency Shader—Alex Ocias Blog) of the virtual hand (Figure 3) according to the biofeedback procedure described in sub-section Experimental Setup. If the transparency index was over a certain threshold, the hand was visible enough to trigger a visuo-tactile event when the sphere approached the hand. In that case the virtual hand on the screen showed a “shaking” animation and the smartphone under the real hand of the subject vibrated. Overall, the SARB is characterized by gamification features (from the challenge to the set of feedback) designed to engage the user in self-regulation activities (Pacholik-Zuromska, 2021) that will be described in next paragraphs.

### Respiratory Biofeedback and Data Acquisition

The SARB was adopted to evaluate two experimental conditions: slow RR and normal RR. The following sub-sections describe how the data were collected and processed for implementing the SARB and assessing the presence and the entity of the expected effects of slow RR.

#### Breathing Data

Breathing data was extracted by analyzing audio signal acquired during the experimental sessions. The procedure aims to detect the current breathing state of the subjects, and their changes: Rest, Inhalation, Exhalation.

The breath states detection was based on the loudness of the signal using an automated custom software (based on C# within a Unity project). The values used depended on this implementation of the SARB system, and they were manually defined by adjusting the values in Avalur (2013). Specifically, we classified the breath events with respect to the maximum amplitude of the recorded breath signal.

A headset was provided to the subjects to be used as an audio recording source. This allowed to comfortably keep a microphone close to the breathing sound source. The headset is a Canyon CORAX Gaming Headset CND-SGHS5, representative of entry level, non-professional devices which might prove affordable for home setups. The experimental setup is positioned in a controlled room to exclude major sources of noise. After acquiring the audio signal, a custom software evaluated current breath states of the subjects: Rest, Inhalation, Exhalation. This step was performed by computing the signal loudness and testing it against a set of threshold values. Starting from the signal loudness, the baseline noise allows to detect the Rest State: *Loudness* < *InhaleMin*. Small amplitude variations determine the Inhalation State: *Loudness* ϵ *[InhaleMin, InhaleMax]*. Big amplitude variations determine the Exhalation State: *Loudness* > *ExhaleThresh*. The thresholds chosen for the present experiment are: 0.05 for *InhaleMin*, 0.1 for *InhaleMax*, 0.3 for *ExhaleThresh*. A different microphone setup might require an adjustment of these values, since they strictly depend on the characteristics of the analyzed signal, which is itself heavily influenced by the audio acquisition factors mentioned above.

Breath frequency detection was performed over audio signal blocks of the duration of 1 s each. This analysis was executed by design at 50 Hz (every 20 ms): this implies an overlap of 980 ms between consecutive audio signal blocks. The sequential steps to detect the breathing frequency were: (i) acquisition of an audio signal block of the duration of 1 s, (ii) calculation of the envelope of the signal representing the loudness (expressed as the root mean square of the raw signal) of the microphone signal multiplied by a scale factor of 10 and the pitch (power spectrum of the signal), (iii) detection of the breathing phases (Rest, Inhalation, Exhalation), (iv) removal of artifacts, (v) computation of the breathing frequency.

Artifact removal (step iv) is required since, despite the controlled setup (headset microphone + controlled room), the recording arrangement for this experiment is still extremely sensitive to background sound and to speech. As a consequence, artifacts had to be removed by filtering the signals and excluding what had to be considered false breathing states triggers. In particular, a rejection procedure was implemented which excluded all the Exhalation and Inhalation state change triggers that were produced by a sound pitch out of the 500–4,000 Hz band. Artifact removal was performed through our custom software solutions, developed in C#.

The exhalation loudness is considerably higher than the inhalation loudness. Therefore, the exhalation event is easier to detect and for each of them a time stamp (*Te*_*t*_) is saved to finally determine the frequency of breath (*Fb*_*t*_):

(1) $$\begin{array}{l} Fb_{t}=\frac{60}{Te_{t}-Te_{t-1}} \end{array}$$

where *Fb* is the new breath frequency at the time *t*+*1*, Te_t+1_ is the time stamp event of exhalation at time *t*+*1* and Te_t_ is the time stamp event of exhalation at time *t* (time in s, breathing frequency in breaths per min).

#### Respiratory Biofeedback

The biofeedback depended on the condition of the task, asking the subjects to keep a “Slow Breathing” rate (about 6 breaths/min) (Schwerdtfeger et al., 2020) or “Normal Breathing” rate (about 14 breaths/min) (Fonkoue et al., 2018).

For both conditions, when a new frequency of breath was detected, it was compared with the target breathing rate (*Fopt*) to produce a value between 0 (transparent) and 1 (opaque) of transparency (*Alpha*):

(2) $$\begin{array}{c} Alpha_{t}=\left\{\begin{array}{cc} \frac{Fopt}{Fb_{t}}, & Fb_{t}>\;Fopt\\ \frac{Fopt}{(2^{*}Fopt)-\;Fb_{t}} & Fb_{t}<\;Fopt \end{array}\right\}\; \end{array}$$

For the success of the task in each trial (fully visualizing the 3D model of the prosthesis before the sphere disappears), the hand transparency (*Alpha*) needs to be higher than 0.8 (Figure 3A). When transparency was lower than 0.8 (Figure 3B) at the end of the trial, the sphere fell down the hole and the task was considered failed. Each trial started with an *Alpha* = 0.5.

During preparatory tests of the initial prototypes of the setup, the quick changes in the hand visibility often constituted a serious obstacle to the subjects' training to perform the task, especially when the sphere was approaching the hand.

Consequently, a facilitation (*f* = 0.05) of the task was introduced to increase the degree of success in case of occasionally breathing rate far from the target during the entire task:

(3) $$\begin{array}{c} Alpha_{t}=\left\{\begin{array}{cc} Alpha_{t}, & Alpha_{t}>\;Alpha_{t-1}\\ Alpha_{t-1}-f & Alpha_{t}<\;Alpha_{t-1} \end{array}\right\} \end{array}$$

If *Alpha* was >0.8 at the end of the trial, the visuo-tactile vibration feedback was generated as co-occurrence of the visual shaking of the virtual hand on the screen and the vibration of the smartphone, placed under the real right hand, as caused by the collision of the sphere and the hand.

To enable such a haptic event, an API was developed for allowing the control of smartphone vibration and to set up wireless communication (based on a local network) between the laptop and the smartphone. This connection was based on a Unity (Windows) desktop app sending to a Java back-end (running on a Tomcat server) a request for a Unity (Android) mobile app that triggers the vibration of the smartphone when the virtual hand-sphere collision happens.

It must be noted that latency is expected when triggering events across a network. Even for a LAN network, latency is usually negatively affected by wirelessly connected components (e.g., the smartphone used for the experiment). Nonetheless, such latency was not noticeable (under 100 ms) when triggering the events required by this experiment, even more so given the slow pace of the tasks.

### In-session Data Collection and Processing

During the experimental sessions, both data collection programs (Unity custom program and BioGraph) were running on the same laptop, allowing for a data synchronization based on the laptop-generated timestamps. The data generated by the Unity software were collected in a text file, named with the ID of the subject and containing the list of breathing events with their time stamps during the experiment. The data collected through the FlexComp Infiniti system were recorded and exported in a text file through the BioGraph software at 2,048 Hz. Downsampling at 256 Hz was performed to allow data synch with the breathing data generated by the Unity software.

The power spectrum of each BVP sequence was reconstructed through the Welch method (eight Hamming windows with 50% overlap). Frequencies in the 0.05 to 0.5 Hz (corresponding roughly to 3 to 30 breath per min) have been considered as generated by respiratory activity, thus the center of the frequency bin with the highest power provides a good estimate of the RR. The RR value, expressed in breaths per min, was then simply estimated by multiplying the obtained frequency by 60.

GSR in each trial was compared for checking potential anticipatory responses to upcoming virtual stimuli (possibly related with the hypothetical different degrees of embodiment in slow and normal breathing conditions): each sequence was normalized as to have a mean value of 0 and a standard deviation of 1, then the value at time 0 was subtracted from each sequence. Normalized sequences were then averaged over trials and subjects for each experimental condition. It must be added that, in RHI studies, skin conductance typically offers information on individual reactions to threatening events (Senna et al., 2014). However, this signal increases to both aversive (Armel and Ramachandran, 2003) and appetitive stimuli (Le et al., 2019): thus, we decided to adopt it to evaluate potential anticipatory reactions to the (uncertain) outcome of the trial, when the hand could vibrate (marking a successful trial) or not.

### Experimental Procedure

#### Instructions and Tasks

##### Session Preparation

Initially, the subjects were asked to wear the (appropriately sanitized) headset and biosensors comfortably. All participants were asked to sit in front of a desk and to place their hands at the sides of a monitor lying (slightly tilted on a foam support toward the subject) on it.

Then, their right hand was placed on the smartphone (the amputees did not wear any prosthesis during the session, thus they placed the right stump on the phone). The position of the phone was marked with tape as a reference for the post-session estimation of the proprioceptive drift.

**Figure 4:**
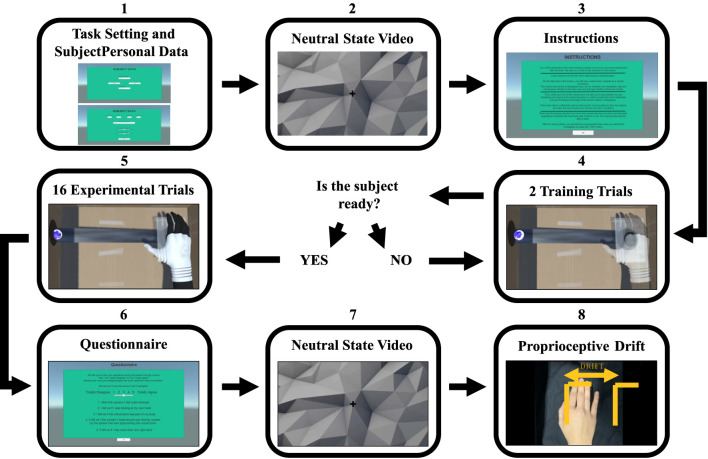
Experimental phases.

After this, the subjects agreed to start the experimental session, allowing the experimenter to begin the acquisition of the respiratory events and the physiological data and to change the Unity scenes (observed through a secondary screen) according to the commands of the participants during the session itself. Figure 4 shows the main Unity scenes and phases of the experimental procedure.

In the first scene (Figure 4, scene 1), the experimenter inserted the subjects' number, set the connection between the laptop and the smartphone through the local network, and chose the breathing condition. In the second scene, the investigator filled the subjects' personal data while reading aloud the different sections to properly transcribe the subjects' answers.

After this, the first instructions scene introduced a 3-min video (Figure 4, scene 2). This video had the goal to induce a neutral mental state before initiating the actual experimental session. The investigator asked the subjects to stay still while fixing the cross in the middle of the screen.

##### Training and Testing Trials

Subsequently, the second instructions scene was read aloud by the experimenter (Figure 4, scene 3), who explained the upcoming short training sample. This scene contained different instructions about the task according to the experimental condition of the ongoing session:

- in the Slow Breathing (low RR) condition, the subjects were asked to maintain the respiratory rate at a slow pace (about 6 breaths/min) to make the virtual hand “materialize” (become visible) enough for feeling the energy of the sphere when it approached the virtual limb,
- in the Normal Breathing (typical RR) condition, the subjects were asked to maintain the respiratory rate at a normal pace (about 14 breaths/min) to achieve the same goal.

In both cases, the subjects were invited to blow on the microphone when they were breathing out. This instruction was given to help the participants in maintaining the expected pace and to produce a sound correctly interpreted by the SARB system.

As described before, by maintaining the right RR of the assigned condition (Slow Breathing or Normal Breathing), during the sliding of the energetic sphere from the hole on the left wall to the hole under the Hannes 3D model, the participants were able to decrease the transparency of the virtual hand to make the virtual hand solid enough to “feel” the energy of the approaching sphere as a vibration. This event meant that the trial was successfully accomplished (Figure 3A). This task was expressed by asking the subjects to “make the hand visible and solid enough to intercept and the sphere and feel its energy.” The duration of each trial was 1 min: the time spent by the sphere to move from the left hole to the right hole on the screen.

Once a training session constituted by two trials (Figure 4, scene 4) was completed, the subjects had to decide to repeat the training or proceed. There was no limit in the repetition of the training trials.

When the participants declared to be ready to start the experimental session, a series of 16 trials started (Figure 4, scene 5), each one based on the 1-min animation and the respiratory biofeedback task described above.

Each trial started after the end of the previous one within the same scene: the sphere disappeared into a hole under the virtual right hand and re-appeared on the left side of the screen. The resulting visuo-haptic events are far less frequent than the ones in typical RHI and VHI studies: this choice depended on the need to perform the biofeedback exercise over an appropriate time to reach the target respiratory pace.

##### Subjective Questionnaire

After completing the experimental trials, the subjective questionnaire scenes appeared instantaneously (Figure 4, scene 6).

The experimenter read aloud the questionnaire instructions, asking the subjects to rate their experience during the session through a score from 1 for “Total Disagreement” to 5 for “Total Agreement” per each statement. Through this, the participants defined how much they disagreed/agreed with the following 14 statements that represented different aspects of virtual hand ownership (items 2, 3, 4) and real hand disownership (items 9, 10, 11) and individual experience—stress (item 1), emotional engagement (item 12), interoceptive intensity (item 13), perception of the relationship between virtual hand visibility and breathing rate (item 14) (Table 1). Control items (5, 6, 7, 8) were included for checking the subject's compliance with the experimental instructions.

**Table 1:** Subjective questionnaire scores (median, Mdn; median absolute deviation, MAD; mean, M; standard deviation, SD).

**N**	**Questionnaire items**	**Slow breathing**				**Normal breathing**				
		**Mdn**	**MAD**	**M**	**SD**	**Mdn**	**MAD**	**M**	**SD**	
1	After this session I feel quite stressed	2	1	2.55	1.26	3	1	3.00	0.93	
2	I felt as if I was looking at my own hand	2	0	2.14	0.71	2	0	1.91	0.53	
3	I felt as if the virtual hand was part of my body	3	0	2.50	0.60	2	0	2.05	0.65	^*^
4	It felt as if the contact I experienced was directly caused by the sphere that was approaching the virtual hand	3	1	2.95	0.90	2	0	2.27	0.83	^**^
5	It felt as if I had more than one right hand	1.5	0.5	1.77	0.92	2	1	1.68	0.72	
6	I felt as if my real hand was turning virtual	2	0	1.91	0.53	2	0	1.73	0.63	
7	I felt as if I could move the virtual hand	2	0	1.95	0.58	2	0	1.82	0.59	
8	It felt as if the contact I experienced came from somewhere between my own hand and the virtual hand	2	1	1.91	0.81	2	0	1.86	0.56	
9	It seemed as if my hand had disappeared	2	0	2.23	0.87	2	1	2.32	0.89	
10	It seemed as if I could not really tell where my hand was	3	0	2.50	0.80	2	1	2.36	0.95	
11	It seemed as if I was unable to move my hand	3	0	2.77	0.75	2	0.5	2.36	0.73	^*^
12	I felt emotionally involved in the situation	3	1	3.05	1.00	3	1	2.95	1.09	
13	I perceived intensely my bodily sensations	3	1	3.14	1.21	3	1	3.18	1.01	
14	I felt the relation between my breath and my virtual hand	3	1	3.18	1.22	4	1	3.55	0.96	

* *, p < 0.05*;

** *, p < 0.01 (Wilcoxon signed-rank test between conditions of Slow Breathing and Normal Breathing)*.

The subjects read silently by themselves each of the 14 statements, divided in 3 scenes, and told the investigator the different scores. To conclude, the experimenter asked the subjects to estimate the duration of the experimental session (in min) for evaluating further potential effects of the breathing condition. The questionnaire was partially adapted to the case of the amputees, referring to their “limb” instead of their “hand.”

##### Proprioceptive Drift Measurement

After collecting the questionnaire answers, the experimenter moved to another instruction scene concerning the final 3-min video to induce a neutral state (Figure 4, scene 7) for restoring the neutral state before measuring the proprioceptive drift (Figure 4, scene 8). Once the video was over, the participants were asked to close their eyes, and the black blanket on the right arm was removed. The reference position of the phone (previously marked by tape) was checked after removing the blanket. If the phone had been moved during the session by more than 5 mm in any direction the following measure of the drift would have been considered unreliable. Otherwise, the researcher marked this position of the phone as final reference position, representing where the phone (thus, the right hand) was during the experiment. After this, the participants were asked to raise their right arm while holding the smartphone and to wave it around to briefly stretch.

Thus, the participants were asked to relocate the smartphone in the perceived initial position, always while keeping the eyes closed. Differently, the prosthesis users only raised their right limb (always with closed eyes) and, after the experimenter removed the smartphone to avoid obstacles, they placed the stump where they felt it was during the session. The estimated position of the phone (which, in the case of the prosthesis users was re-placed by the experimenter under the relocated stump) was marked with tape to facilitate the measurement of the drift from the reference position, previously marked with tape too.

**Figure 5:**
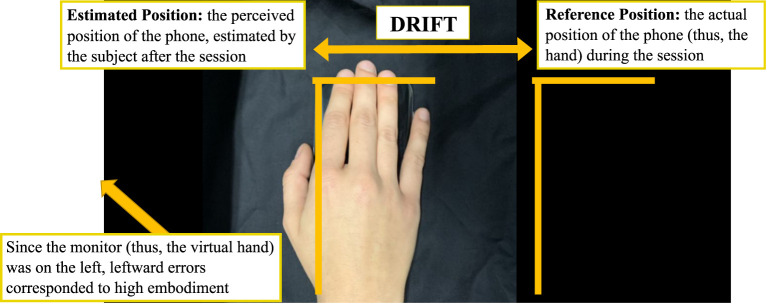
Proprioceptive drift measurement—only the lateral error from the actual phone position (reference position) was considered.

The lateral distance between the reference position of the phone and the one estimated by the participants were measured by the experimenter, together with the direction of the deviation (Figure 5). To measure the drift we assumed the reference position of the phone during the session as 0 point of a continuous horizontal scale with negative values to the left (toward the virtual hand) and positive to the right.

This strategy to estimate a proprioceptive drift was specifically devised for this setup, considering how it could facilitate this part of the experiment in home training sessions: marking with tape the position of a rectangular object representing the hand position is far easier than performing the same operation with the hand itself as a reference.

After this, the sensors, the headphones, and the blankets were removed, and the subjects were free.

#### Experimental Design and Statistical Analysis

In a within-group experimental design, all participants performed the tasks under Slow Breathing and Normal Breathing conditions. Each condition was experienced by the participants in different days with max 14 days between sessions. The order of sessions was counterbalanced, by also accounting for gender and age (as much as possible) to compose the resulting two sub-groups: 11 (3 females) subjects (Age, mean ± SD: 27.6 ± 2 years) who were presented the Slow Breathing condition in first session and the Normal Breathing condition in second session, and 11 (3 females) subjects (Age, mean ± SD: 27.2 ± 2.8 years) who were presented the condition in the opposite order. Following the exploratory function of this preliminary study, we used two-tailed tests for observing potentially significant differences in both directions.

The questionnaire data were analyzed via Wilcoxon signed-rank test with the breathing condition—Slow Breathing vs. Normal Breathing—as factor. The scores of each item were compared. Further comparisons were based on average scores per sub-set of questionnaire items as global indices of ownership, disownership, and control as in Pyasik et al. (2020).

Session time estimation and proprioceptive drift were analyzed via paired samples *t-*test with breathing condition as a factor.

The frequency of respiratory events was analyzed to assess the feasibility of this setup by evaluating the participants' capability to control their own number of breaths per trial (being each trial 1-min long) according to the instructions. The breathing condition being the factor, the breaths per trial were analyzed via *t*-test. The same comparison was performed for the number of successful trials as a performance measure (the number of trials in which the subjects made the virtual hand vibrate).

GSR signals have been analyzed to identify possible time segments for which responses differed significantly from the end-point value, implying a possible anticipatory response. Given the normalization described in In-session Data Collection and Processing, this analysis consisted simply in testing grand-averages across subjects and trials to identify time segments with median values different from zero. Specifically, a Wilcoxon signed-rank test for zero median has been conducted on the skin conductance signal. In order to prevent possible false positives due to slow signal drift, this analysis has been limited to the last 10 s of recording before each visuo-tactile event.

All analyses were performed using JASP (https://jasp-stats.org) (Love et al., 2019), R (https://www.r-project.org), and Matlab (MathWorks, Inc.), and *p* < 0.05 was considered significant.

The next section focuses on the significant results in all comparisons, with statistically relevant information like the effect size (Cohen's d for the parametric tests, rank-biserial correlation for the Wilcoxon signed-rank test) (Kerby, 2014) and the test assumption check (only Shapiro-Wilk test of normality for repeated measures parametric tests with one 2-level independent variable).

## Experimental Results

### Virtual Hand Ownership

In the Slow Breathing condition, participants reported stronger feelings that the virtual hand was part of their body (item 3, with *W* = 106 and *p* = 0.035), that the contact experienced was directly caused by the sphere that was approaching the virtual hand (item 4, with *W* = 122 and *p* = 0.003), and that they were unable to move their own right hand (item 11, with *W* = 96 and *p* = 0.022), compared to the Normal Breathing condition (see Table 1). Rank-biserial correlation was used to estimate the effect size and the related confidence interval, respectively with values of: (item 3) 0.559 and 95% CI [0.074, 0.83], (item 4) 0.794 and 95% CI [0.482, 0.927], (item 11) 0.6 and 95% CI [0.117, 0.853].

Significant differences were found between the control (5, 6, 7, 8) items average score and, respectively, the ownership (2, 3, 4) items average score (*W* = 220.5 and *p* < 0.001 in Slow Breathing, *W*= 195 and *p* = 0.027 in Normal Breathing) and the disownership (9, 10, 11) items average score (*W*= 206.5 and *p* < 0.001 in Slow Breathing, *W*= 223.5 and *p* = 0.002 in Normal Breathing). Rank-biserial correlation was used to estimate the effect size and the related confidence interval. For the ownership-control comparison: 0.909 and 95% CI [0.776, 0.965] in Slow Breathing, 0.542 and 95% CI [0.128, 0.794] in Normal Breathing. For the disownership-control comparison: 0.967 and 95% CI [0.912, 0.988] in Slow Breathing, 0.767 and 95% CI [0.489, 0.903] in Normal Breathing.

A significant difference (*W* = 153.5 and *p* < 0.001) was also found between the ownership average scores in each breathing condition (Table 2). According to rank-biserial correlation, the effect size and the related confidence interval are respectively 0.795 and 95% CI [0.508, 0.923].

**Table 2:** Average scores of items on ownership, control, disownership (median, Mdn; median absolute deviation, MAD; mean, M; standard deviation, SD).

**Questionnaire items average scores**	**Slow breathing**				**Normal breathing**				
	**Mdn**	**MAD**	**M**	**SD**	**Mdn**	**MAD**	**M**	**SD**	
Ownership (items 2, 3, 4)	2.67	0.333	2.53	0.54	2.17	0.167	2.08	0.52	^**^
Control (items 5, 6, 7, 8)	1.88	0.375	1.89	0.45	1.75	0.25	1.77	0.42	
Disownership (items 9, 10, 11)	2.67	0.5	2.5	0.66	2.33	0.5	2.35	0.75	

** *, p < 0.01 (Wilcoxon signed-rank test between conditions of Slow Breathing and Normal Breathing)*.

Overall, the participants estimated the total duration of the task (16 min) as: 11.55 ± 5 min in Slow Breathing, 12.77 ± 4.03 min in Normal Breathing (no significant difference between conditions).

**Figure 6:**
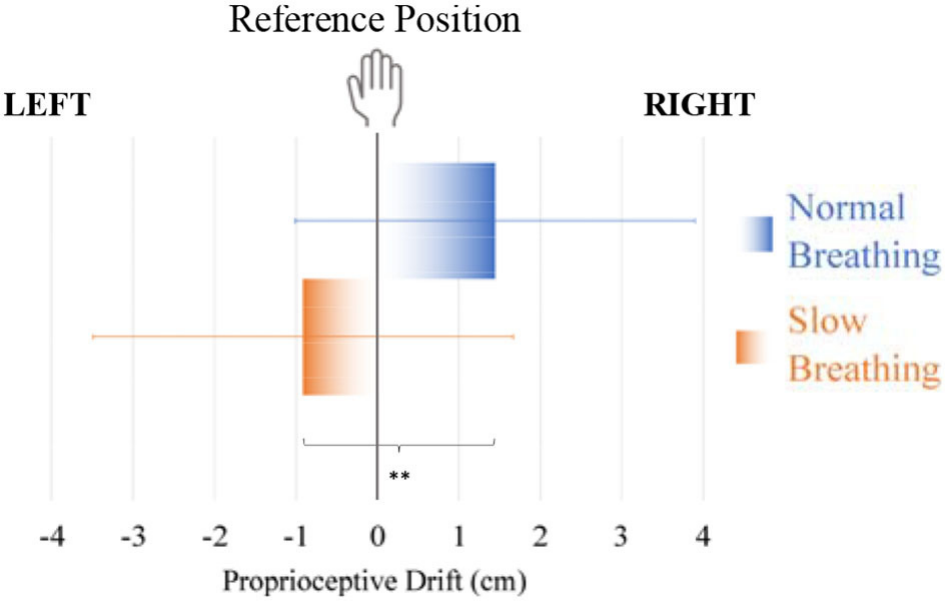
Comparison of proprioceptive drift (cm) from the reference position of the hand (0) in conditions of Slow Breathing and Normal Breathing, with means and standard deviations. **p < 0.01 (pairwise *t-*test between conditions of Slow Breathing and Normal Breathing).

Considering the proprioceptive drift, no subject moved the phone during the session (before the drift estimation) by more than 5 mm in any direction: thus, all measures were included in our analysis. According to the collected data, the breathing condition significantly affected the proprioceptive drift: *t*(21) = −3.558, *p* = 0.002, *d* = −0.759, CI [-1.23, −0.276] (Figure 6). The drift comparison between Slow Breathing and Normal Breathing successfully passed the Shapiro-Wilk test of normality, with 0.975 (*p* = 0.824). The participants estimated the position of the smartphone, i.e., their right hand, to the left of its actual location (averagely by 0.91 ± 2.58 cm) and closer to the monitor i.e., the virtual hand, in the Slow Breathing condition. The same estimation was to the right of its actual location (averagely by 1.45 ± 2.45 cm) in Normal Breathing condition.

The analysis of GSR (planned as in Experimental Design and Statistical Analysis) shows that, in the considered time window, the measured values are significantly different from the end value at the 0.05 significance level only in Normal Breathing condition (between 1.7 s and 1.3 s before the end of the trial).

### SARB Feasibility

**Figure 7:**
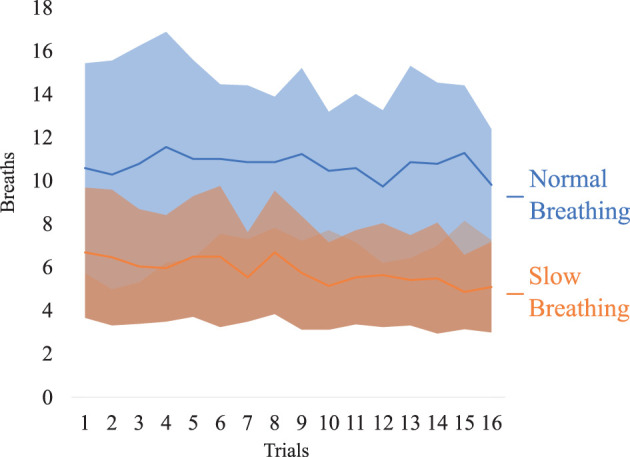
Means (continuous lines) and standard deviations (shaded areas) of breaths per trial in conditions of Slow Breathing and Normal Breathing, along the 16 trials (1 trial per min).

Figure 7 highlights how the subjects followed the instructions for each condition according to the data collected through the microphone and processed by the custom Unity software. No significant difference can be found considering both the breathing condition and the trial repetition as factors. However, in the Slow Breathing condition participants maintained 5.8 ± 2.5 breaths per trial, overall. This value was significantly lower than the Normal Breathing condition, 10.7 ± 2.6 breaths per trial, as expected: *t*(21) = −8.382, *p* < 0.001, *d* = −1.787, CI [−2.459, −1.098]. The comparison successfully passed the Shapiro-Wilk test of normality, with 0.951 (*p* = 0.335).

Additionally, an exploratory analysis of BVP values was performed for extracting the frequency of respiratory events and comparing it to the data collected by the Unity software, showing no significant difference between them in each breathing condition.

Before moving on to the experimental session, 4 participants asked to repeat (1.75 ± 0.5 times, by average) the training session in Slow Breathing condition. Three of these subjects needed to repeat (1.33 ± 0.58 times, by average) the training session in Normal Breathing condition too. Then, over 16 total trials, the participants were able to make the virtual hand “shake” (when, at the end of each trial, the transparency index *Alpha* > 0.8) by average (without significant differences): in 10.77 ± 4.94 trials under Normal Breathing condition, and in 9.36 ± 3.44 trials under Slow Breathing condition.

### Preliminary Test With Users of Prostheses

Both the users of upper limb prostheses involved in this study followed our instructions in terms of breath control. In Slow Breathing condition, one subject (who repeated the training session two times) had a mean 6.3 ± 2 breaths per trial and the other (one repetition of the training) had 4.94 ± 2.5 breaths per trial. In Normal Breathing condition, they respectively had (after repeating two times and one time the training) a mean number of breaths per trial of 11.31 ± 2.5 and 13.19 ± 3.02. About task performance: in Slow Breathing condition they respectively achieved 8 and 12 successful trials over 16, and in Normal Breathing condition 15 and 11. These preliminary tests with two amputees suggested the potential for implementing home-based embodiment training systems with affordable solutions for respiratory biofeedback.

Overall, their questionnaires showed higher scores than the individuals interviewed for the main study, surpassing the middle value of the 5-point Likert-type scales. The scores (Table 3) demonstrate medium-high values of ownership and engagement with a minimal stress. The session time estimation reported by each subject in both conditions was lower than the actual 16 min of trials, respectively: 10 min and 15 min in Slow Breathing, 5 min and 10 min in Normal Breathing.

**Table 3:** Post-trials subjective evaluation questionnaire scores reported by the two users of upper limb prostheses.

**N**	**Questionnaire items**	**Slow Breathing**		**Normal Breathing**	
		**Subject** **A**	**Subject** **B**	**Subject** **A**	**Subject** **B**
1	After this session I feel quite stressed	1	2	1	2
2	I felt as if I was looking at my own limb	4	3	4	3
3	I felt as if the virtual limb was part of my body	5	2	4	3
4	It felt as if the contact I experienced was directly caused by the sphere that was approaching the virtual limb	5	4	3	4
5	It felt as if I had more than one right limb	1	1	2	1
6	I felt as if my real limb was turning virtual	5	1	3	1
7	I felt as if I could move the virtual limb	3	2	2	3
8	It felt as if the contact I experienced came from somewhere between my own limb and the virtual limb	1	2	1	4
9	It seemed as if my limb had disappeared	1	1	5	2
10	It seemed as if I could not really tell where my limb was	1	1	1	3
11	It seemed as if I was unable to move my limb	5	3	5	2
12	I felt emotionally involved in the situation	4	3	4	3
13	I perceived intensely my bodily sensations	5	3	5	3
14	I felt the relation between my breath and my virtual limb	5	3	5	3

The proprioceptive drift of each subject tended in both conditions toward the virtual hand, respectively: 3 cm and 4.7 cm in Slow Breathing, 3 cm and 2.5 cm in Normal Breathing.

## Discussion

This study provides preliminary evidence of how self-regulation techniques (via respiratory control) can increase the processes of body ownership underlying the embodiment of a virtual right hand. It also highlights the feasibility of the implementation of SARB within the boundary of remote studies.

Our results (questionnaire scores, proprioceptive drift) indicated that our slow breathing biofeedback (vs. normal breathing) may improve the ownership process, i.e., increasing the sensations that the virtual hand was part of the subject's body and that the vibration experienced by the subject was caused by the sphere on the screen. While both aspects are directly connected to the embodiment process (which depends on the perceived relation between self and body), the last one could also be related to the feeling of presence: the experience of “being there” in a mediated environment (Riva et al., 2003).

Thus, we can infer that the Slow Breathing condition made the participants feel that their body was extended (through the artificial limb) into the digital on-screen component of the SARB environment, when compared to Normal Breathing condition. Such an effect needs further investigation while studying the role of Slow Breathing in improving presence and avatar control, also considering the relationships between embodiment and presence (Rosa et al., 2020). Interestingly, the assessment of certain subjects' feeling (reported through questionnaire responses and spontaneous remarks) of being unable to move their own right hand, unveils a side-effect of Slow Breathing in terms of disownership.

The SARB setup was effective in monitoring individuals' breathing, processing the respiratory rate and providing the desired feedback to the users. The subjects were able to follow the instructions properly, generating two different condition-specific breathing rates. However, we noticed that the subjects tended to have a lower respiratory rate than the target, and their performance in terms of successful trials was quite variable across the subjects (highlighting how maintaining an appropriate RR to trigger the vibration can become complex to manage). These observations point at the need of a task re-design for facilitating the execution of the biofeedback training, especially considering the high inter-subject variability of the successful trials in this study (pointing at potential usability issues for certain participants) and the potential effects of workload on body ownership measures (Qu et al., 2021).

Furthermore, such a re-design should also focus on improving the user engagement, since the setup was just moderately able to stimulate the participants through its current gamification features. Indeed, most questionnaire scores did not overcome the middle point of 3 in the 5-point Likert-type scales, and anticipatory responses were just weakly detected in GSR patterns only under Normal Breathing condition. This could depend on the fact that our implementation of SARB was based on a limited number of tactile events: 16 occurrences (1 per min) just in case the person performs the task correctly in each trial. In classic RHI studies, these stimulations are more frequent and numerous in a shorter time, making most people experience the illusion within the first minute of the session (Kalckert and Ehrsson, 2017). Furthermore, our SAR environment was probably less immersive than the ones used in most VHI settings, affecting both the strength and the variability of the embodiment measures (in particular the proprioceptive drift). VHI studies typically provide a strong perceptual continuity between computer-generated body parts (hand and arm) of the subject within the same immersive context, with advantageous effects on the embodiment measures. However, our goal was to observe if these measures were significantly different in Slow Breathing condition and Normal Breathing condition within the same setting, and this was confirmed by our preliminary data. In any case, the role of the attentional effects of respiratory control needs to be also considered by, for example, separating focus-attention on breathing from the feedback-control components.

Considering its methodological value, our SARB-based procedure can be considered an original addition to the heterogenous family of RHI studies (Riemer et al., 2019). Specifically, SARB can constitute an affordable home training system for the embodiment, but it needs further design improvements, possibly exploiting more game-based features to engage the users. This can be a promising strategy, especially if validated through long-term home experiments (Garske et al., 2021), even within wider and engaging digital health protocols (Winkler et al., 2019). The opportunity of using this kind of approach for developing novel strategies to investigate psychopathological conditions will also be considered, especially when the interoceptive processes are involved, as in Grynberg and Pollatos (2015), for example.

Being aware of the limitations of this initial study, we are anyway encouraged by the current preliminary results: SARB constitutes a viable approach in implementing a self-regulation of psychophysiological states to promote the embodiment of an artificial limb through a Slow Breathing condition. Furthermore, this study offered the opportunity of preliminarily testing our hypothesis and our setup before proceeding with further laboratory investigations and with extensive home data collection sessions.

Accordingly, the dual value of the investigation presented in this paper suggests two possible directions for the next steps of this research (envisioning their subsequent convergence too).

- Psychophysiological studies (in laboratory) would allow to investigate specifically the EEG correlates of the virtual hand embodiment (Kanayama et al., 2021) in a SARB setting (using chest belts to precisely monitor RR). A potential target could be the study of Slow Cortical Potentials (SCPs, 0.01–0.1 Hz) (Hinterberger et al., 2019) correlated with the heartbeat and the respiration cycle, thought to be also implicated in stimuli integration (Northoff, 2016).
- User experience studies (in laboratory and in remote contexts) on the SARB setting would initially help to improve the usability of the setup, making the task easier and more engaging (possibly personalizing the target RR through adaptive and co-adaptive features) for the participants in upcoming remote online sessions (even as daily game-like training) (Ratcliffe et al., 2021). The visual scene will be improved with further graphic details to achieve a more compelling experience (e.g., substituting the black area around the right hole with a more realistic texture). Next studies will include amputees exploiting the respiratory biofeedback strategy for training the embodiment of artificial limbs.

Extending the sample size will allow for controlling factors based on the subjects' traits and habits (e.g., playing videogames or sports, smoking). Importantly, their body image and interoceptive awareness should be assessed (Mehling et al., 2012) alongside the personality features (Burin et al., 2019).

Further investigations must also demonstrate if the effects of the SARB-based training persist over time, and if an actual generalization of the embodiment of the 3D model of a prosthesis can be observed for the actual device (Laffranchi et al., 2020), possibly exploiting the latter in game-like XR remote trainings designed to engage the users. This solution (alongside with the adoption of ecologically valid settings as in neuroergonomics research) (Dehais et al., 2020) could counter the apparent lack of RHI-susceptibility in subjects who feels prosthetic limbs ownership mainly when the devices are used in daily life (Zbinden and Ortiz-Catalan, 2021).

As discussed above, this kind of RHI-resistance was found in meditators (Xu et al., 2018). However, differently from previous studies, we explored the embodiment as a process affected by an active respiratory control within a biofeedback protocol instead of just presenting a typically passive RHI-like test without asking to perform any respiratory task. Accordingly, we hypothesize that the fine control of RR matured through meditation practices could be advantageous in SARB procedures, possibly working as a preparatory activity to our respiratory biofeedback for embodiment training—especially for patients attending telerehabilitation protocols and amputees waiting for their prosthesis.

## Conclusion

This pilot study presented a novel, affordable strategy for empowering the feeling of owning a virtual hand through an individual self-regulation method based on a respiratory control aiming at slow breathing. The design of the setting, targeting remote studies, showed the feasibility of implementing such a system with common devices owned by users like a computer, a monitor, a smartphone, and a microphone. Thus, this proof of concept offered a preliminary (methodological and technological) background for developing novel user-centered strategies in research and design to facilitate the embodiment of artificial limbs.

## Data Availability Statement

The dataset generated for this pilot study may be available to readers upon reasoned request to the corresponding author.

## Ethics Statement

The study followed an experimental protocol, involving human subjects without sensory, cognitive, and neuromotor impairments (exclusion criteria referred to any neurological condition affecting the capability of the individual to perform the tasks). The protocol was reviewed and approved (March 16th, 2020) by the Ethical Committee of Liguria Region in Genoa, Italy (IIT ADVR TELE01, Register Number: 229/2019 - ID 4621). All subjects provided their own written informed consent to participate in this investigation and to publish any anonymized image and data collected by the researchers.

## Author Contributions

GB conceived the research hypothesis and the interaction paradigm adopted in this study, reviewed literature on related topics, designed the task and the visuo-tactile feedback, defined the experimental design, and the research methodologies. AM, GC, MdZ, JT, LA, NB, MF, DM, and MB contributed to improve the experimental design, the interaction paradigm, and the research methodologies. GB and MF performed preparatory activities to implement the paradigm and the investigation, including the sample size calculation. NB, MF, DM, ND, CF, MB, EG, ML, and LDM contributed to define the research perspective according to its potential applications. AM and GC designed and developed the experimental setup and the interactive environment. AM devised and implemented the systems enabling the breath detection and the spatially augmented respiratory biofeedback. GB and GC recruited the subjects and managed the experimental sessions with data collection. GB analyzed the data and wrote the first draft of the paper, subsequently improved by AM, GC, and MdZ. MdZ, JT, and LA performed further data analyses to check additional results. GB, GC, and NB worked on figures and graphs. Finally, all authors worked on the results interpretation and on the final manuscript writing, and they read and approved the submitted version.

## Conflict of Interest

The authors declare that the research was conducted in the absence of any commercial or financial relationships that could be construed as a potential conflict of interest.

## Publisher's Note

All claims expressed in this article are solely those of the authors and do not necessarily represent those of their affiliated organizations, or those of the publisher, the editors and the reviewers. Any product that may be evaluated in this article, or claim that may be made by its manufacturer, is not guaranteed or endorsed by the publisher.

## Abbreviations

AR: augmented reality
BVP: blood volume pressure
EEG: electroencephalography
GSR: galvanic skin response
HR: heart rate
MR: mixed reality
RHI: rubber hand illusion
RR: respiratory rate
SAR: spatial augmented reality
SARB: spatially augmented respiratory biofeedback
SCP: slow cortical potential
VHI: virtual hand illusion
VR: virtual reality
XR: extended reality.
